# Bystander cardiopulmonary resuscitation training in primary and secondary school children in China and the impact of neighborhood socioeconomic status: A prospective controlled trial

**DOI:** 10.1097/MD.0000000000012673

**Published:** 2018-10-05

**Authors:** Hui Li, Xu Shen, Xia Xu, Yan Wang, Lihua Chu, Jialian Zhao, Ya Wang, Haihong Wang, Guohao Xie, Baoli Cheng, Hui Ye, Yaqi Sun, Xiangming Fang

**Affiliations:** a Department of Anesthesiology, The First Affiliated Hospital, School of Medicine, Zhejiang University, Hangzhou; b Department of Anesthesiology, Jiaxing First Hospital of Zhejiang Province, Jiaxing; c Department of Anesthesiology, Lihuili Hospital, Ningbo Medical center, Ningbo; d Department of Anesthesiology, The Children's Hospital, Zhejiang University School of Medicine, Hangzhou, Zhejiang; e Department of Anesthesiology, Sir Run Run Shaw Hospital Affiliated to Medical College of Zhejiang University, Hangzhou, China.

**Keywords:** bystander CPR training, China, out-of-hospital cardiac arrest, primary and secondary school, SES

## Abstract

**Background:**

The World Health Organization (WHO) has endorsed school bystander cardiopulmonary resuscitation (CPR) training programs. But related researches in China are limited. Therefore, we conducted this study to assess bystander CPR training in school children in China and the impact of neighborhood socio-economic status (SES) on.

**Methods:**

A total of 1,093 students from seven schools in Zhejiang province participated in this study. Theoretical and practical bystander CPR training were conducted in instructor-led classes. Students completed a 10-statement questionnaire before and after training, and then underwent a skills assessment during a simulated basic life support (BLS) scenario. Subgroup analyses were stratified according to neighborhood SES.

**Results:**

Before training, most students (72.83%) had a strong desire to learn bystander CPR and share with others. After training, bystander CPR theory was significantly improved (*P* < .01), and 92.64% students reached an 85-100% performance rate in a simulated BLS scenario. Students from low-SES neighborhoods had less pre-training knowledge of bystander CPR (*P* < .01). However, their performance was similar with students from higher-SES neighborhoods on the post-training questionnaire and the skills assessment, and better among students aged 13–14 years.

**Conclusion:**

School children in China have a poor pre-training knowledge of bystander CPR. However, with training, there was a significant improvement in the basic theory and skills of CPR. Bystander CPR training efforts should be targeted to Chinese primary and secondary school children, especially in low-SES neighborhoods.

## Abstract 2

Supplemental Digital Content is available in the text

## 1 Introduction

Early and effective bystander cardiopulmonary resuscitation (CPR) has become the most important predictor of survival and long-term quality of life in out-of-hospital cardiac arrest (OHCA) patients,^[1–3]^ as emergency-medical-services personnel may not arrive in time to prevent neurological damage. Importantly, studies show that bystanders who have received previous CPR training are most likely to perform CPR at the time of an OHCA.^[4,5]^

Primary and secondary school children are an ideal target audience for bystander CPR training. The World Health Organization has endorsed school bystander CPR training programs,^[6–8]^ which has been successfully implemented in many countries.^[9–12]^ Studies on bystander CPR training for primary and secondary school children in China are limited; however, evidence suggests that effective bystander CPR training programs in Chinese schools could have long-term health benefits for the population.^[13–15]^

Neighborhood socioeconomic status (SES) is a well-known determinant of health outcomes, the incidence of some diseases, and mortality.^[16,17]^ Recent studies demonstrate that individuals from low-SES neighborhood, characterized by lower levels of education and income, have less knowledge of bystander CPR and a lower probability of initiating bystander CPR compared to individuals from higher-SES neighborhood.^[18,19]^ No data currently describe the impact of neighborhood SES on bystander CPR training in school children.

The objectives of the current study were to assess bystander CPR training in school children in China and the impact of neighborhood SES on.

## 2 Materials and methods

### 2.1 Trial design

A prospective controlled trial was conducted in seven schools in Zhejiang province. This study was approved by the Ethics Committee of The First Affiliated Hospital of Zhejiang University and registered in Chinese Clinical Trial Register, and the registration number is ChiCTR-HOC-16009680.

### 2.2 Bystander CPR training program

#### 2.2.1 Theoretical bystander CPR

Theoretical bystander CPR education was conducted in instructor-led classes using a multimedia format and a brief video tutorial demonstrating bystander CPR. The course content was based on the 2015 European Resuscitation Council Guidelines^[20]^ and emphasized the importance of bystander CPR, recognition of cardiac arrest, and emergency procedures.

#### 2.2.2 Practical bystander CPR

Practical bystander CPR training was held immediately after theoretical teaching and was conducted in instructor-led one-on-one classes using Laerdal Little Anne training manikins. Five students were assigned into 1 group according to their student numbers. And then each group was randomly assigned to 5 instructors. The course was conducted based on the guidelines from 2015 European Resuscitation Council and lasted until all the participants were capable of performing CPR.

Subsequently, students participated in a simulated basic life support (BLS) scenario. Skills were assessed using a scoring sheet (Supplementary Appendix A) developed from the 2015 European Resuscitation Council Guidelines and the Cardiff Test for BLS and Automated External Defibrillation Version 3.1: Assessment Guidelines.^[7,20]^ The assessment included evaluation of the consciousness, calling for emergency help, performing chest compression, and airway management.

### 2.3 Pretraining and post-training questionnaires

Students independently completed a 10-statement questionnaire that was distributed and collected by specified personnel before and after theoretical bystander CPR training. Questionnaires (Supplementary Appendix B) were designed according to previous research,^[21]^ while considering the actual situation in China. These questionnaires collected demographic information and assessed each student's willingness to learn first aid and their level of bystander CPR knowledge. Each question was scored on a 10-point scale, with a maximum score of 100 possible for the whole questionnaire.

### 2.4 Participants

Seven schools in 4 cities in Zhejiang province, China participated in this study. The 4 cities were from different socioeconomic regions. Primary and secondary school children from the fifth and sixth grade and the first and second grade, respectively, were included. Physically disabled or injured students are excluded. Course instructors were medical students or anesthesiologists from Zhejiang University who had successfully completed the cognitive and skills evaluation in accordance with the curriculum of the American Heart Association BLS instructor Program.

### 2.5 Study outcomes

The primary outcome of the investigation was the correct rate of CPR knowledge-related items. Secondary outcomes were the impact of neighborhood SES and age on the training.

### 2.6 Statistical analysis

Statistical analyses were performed using SPSS 17.0 for Windows (SPSS Inc, Chicago, IL). Categorical variables were presented as percentages and median (Q1, Q3), and were analyzed using the chi-squared test. Between-group differences were evaluated using *t* test for normally distributed variables and the Mann–Whitney *U* test for non-normally distributed variables.

Subgroup analyses stratified according to neighborhood SES was performed. According to Chiang et al, neighborhood SES was assessed based on the average price of real estate in the administrative districts where the schools were located.^[22]^ Seven schools from 6 administrative districts were included in this study. The 2 districts with the lowest average price of real estate were classified as low-SES neighborhoods, and the other districts were classified as higher-SES neighborhoods. *P* < .05 was considered statistically significant.

## 3 Results

### 3.1 Demographic characteristics

A total of 1093 students from 4 primary (492 students) and 3 secondary (601 students) schools in Zhejiang, China were enrolled in this study. Initially, a total of 8 schools in Zhejiang province were selected to participate in this study. Among these, 7 schools indicated a strong motivation to adopt a bystander CPR training curriculum (87.5%). One school was concerned that students would incorporate bystander CPR training into their games, creating potentially dangerous situations. Consequently, we emphasized that bystander CPR is only applicable to the person with cardiac arrest in emergency situations. No student suffered physical discomfort or injury during training.

The demographic characteristics of students who participated in this study are presented in Table 1. Among these, 990 (90.58%) and 1079 (98.72%) students completed the pretraining and post-training questionnaires, respectively.

**Table 1:**
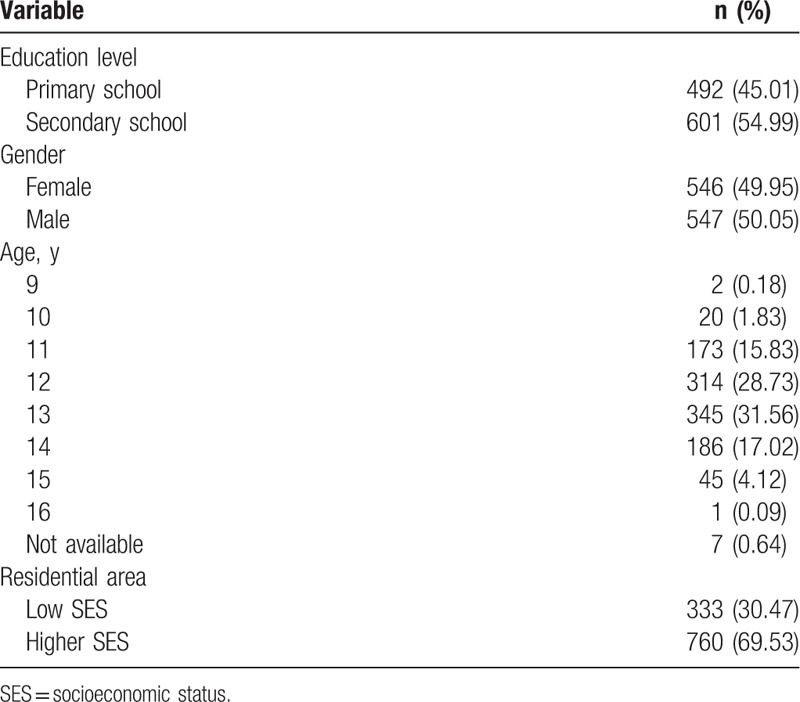
Demographic data (n = 1093).

### 3.2 Pretraining questionnaires

Before bystander CPR training, 235 respondents (23.74%) were unfamiliar with CPR. Among the 746 (75.35%) respondents who were familiar with CPR, 326 (43.70%) respondents had seen or heard of CPR through television, 165 (22.12%) respondents from the internet, and 255 (34.18%) respondents via other sources. The vast majority (923; 93.23%) of respondents had never participated in any CPR-related training. Assuming the respondents had mastered bystander CPR skills, 721 (72.83%) were willing to share their knowledge with others, including relatives, friends, or classmates (Table 2).

**Table 2:**
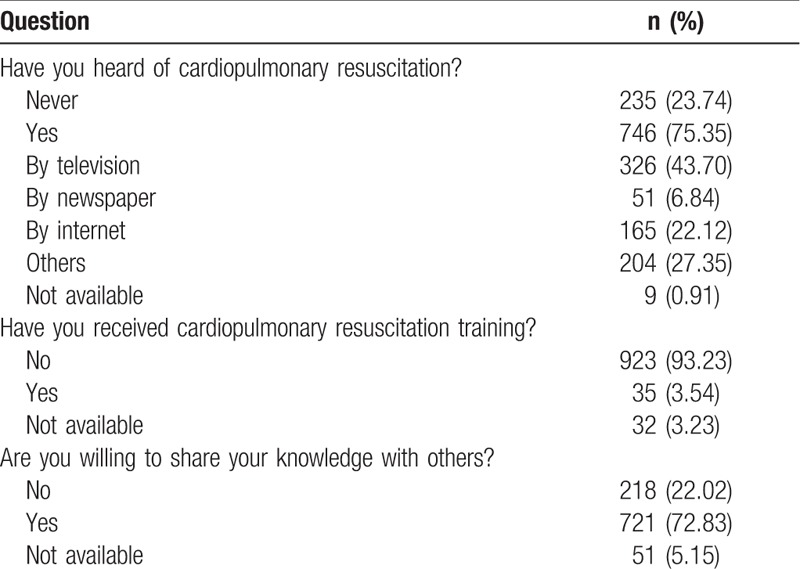
Pretraining questionnaire: attitudes and practical experience of bystander cardiopulmonary resuscitation.

In assessing the bystander CPR knowledge of respondents before training, 358 (36.16%) and 478 (48.28%) respondents chose the correct method to evaluate responsiveness and respiratory movement, respectively. Furthermore, 474 (47.88%) respondents knew the hand placement for compression; 519 (52.42%) and 462 (46.67%) respondents knew the posture of hand and arm for compression. Only 75 (7.58%), 81 (8.18%), and 286 (28.89%) respondents chose the correct compression depth, rate, and ratio of compression to artificial respiration, respectively (Table 3).

**Table 3:**
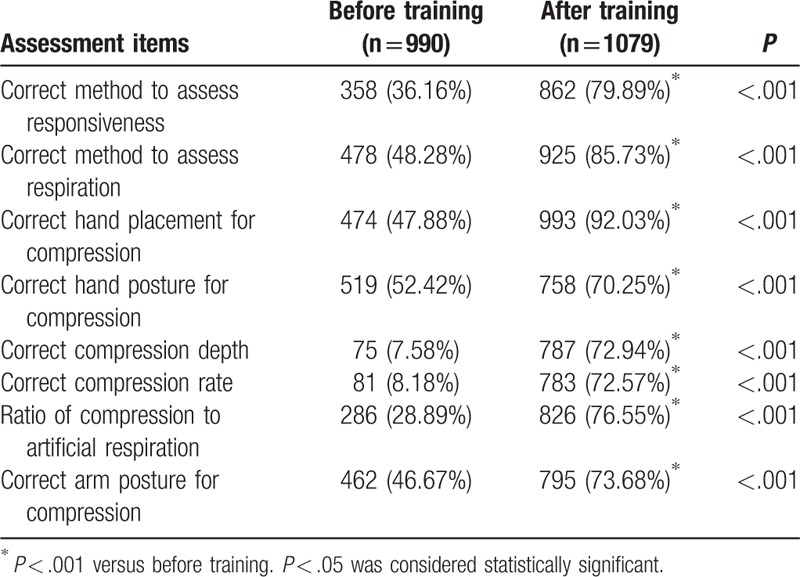
Impact of training on knowledge of bystander cardiopulmonary resuscitation.

### 3.3 Post-training questionnaire

After the bystander CPR training program, significantly more respondents chose the correct method to evaluate responsiveness (79.89% vs. 36.16%, *P* < .001) and respiratory movement (85.73% vs. 48.28%, *P* < .001) compared to pretraining. There was a significant increase in the number of respondents who chose the correct hand placement (92.03% vs. 47.88%, *P* < .001), hand posture (70.25% vs. 52.42%, *P* < .001), and depth (72.94% vs. 7.58%, *P* < .001), rate (72.57% vs. 8.18%, *P* < .001) for compression, and ratio of chest compression to artificial respiration (76.55% vs. 28.89%, *P* < .001) (Table 3).

### 3.4 BLS skills

The students from 5 schools participated in the BLS scenario. When assessing BLS skills and considering students with an 80% performance rate for each skill, 93.66% (458/489) of students called the manikin loudly, shook it gently to evaluate responsiveness, and contacted the Chinese medical emergency telephone number (120) for help; 98.16% (480/489) of students could put the manikin in the recovery position; 98.57% (482/489) of students could provide compressions with the correct hand placement; 96.73% (473/489) and 95.50% (467/489) of students used the correct compression rate and depth; 90.59% (443/489) of students performed compressions with the correct posture; 94.89% (464/489) of students were aware of the airway opening, and 96.93% (474/489) of students ventilated correctly; 91.00% (445/489) of students knew performing 30 chest compressions followed by 2 breathes. Overall, 92.84% (454/489) of students performed bystander CPR proficiently.

When scoring bystander CPR proficiency on a scale of 0 to 100 points, considering 100 points as the most proficient, 478 (97.75%) students scored >77 points, and 401 (82.00%) students scored >90 points (Table 4).

**Table 4:**
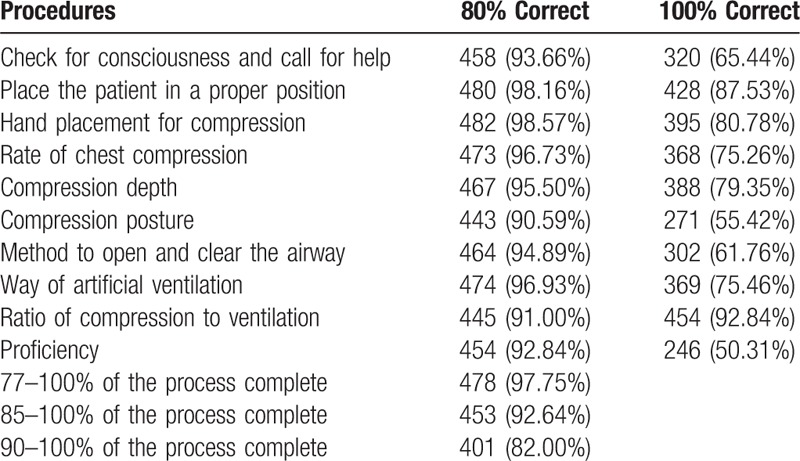
Post-training skills assessment (simulated basic life support scenario) (n = 489).

### 3.5 The impact of neighborhood SES and age on training

Significantly more students from the low-SES neighborhoods were unfamiliar with CPR (44.74% vs. 13.09%, *P* < .001) and were unwilling to share the acquired bystander CPR knowledge (27.91% vs. 20.85%, *P* < .05) compared with the students from the higher-SES neighborhoods (Table 5).

**Table 5:**
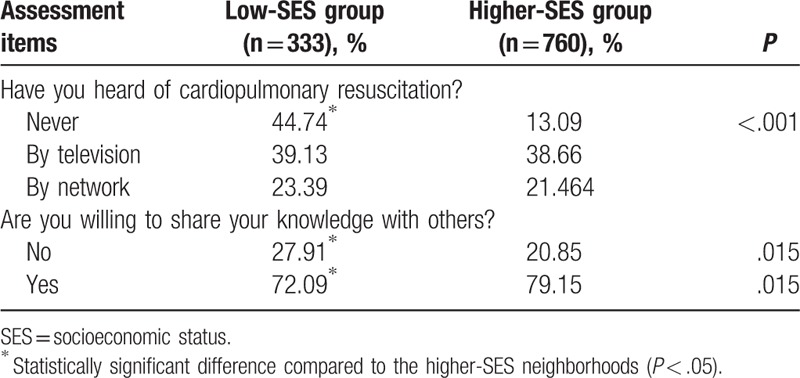
Pretraining experience and attitudes to bystander cardiopulmonary resuscitation stratified according to neighborhood socioeconomic status.

Regardless of age, students from the low-SES neighborhoods scored worse on the pretraining questionnaire. Exceptions were scores on questions related to compression depth, rate, and the ratio of compression to artificial respiration. Scores for the latter items were low among students in both groups, and there were no significant differences (Table 6). On the post-training questionnaire, students from the low-SES neighborhoods who were 13 to 14 years old performed better, and those who were 11 to 12 years had a similar performance on most assessment items compared to students from the higher-SES neighborhoods. Across all age groups, students from the low-SES neighborhoods did as well as students from the higher-SES neighborhoods on the total scores of the BLS skills assessment (Table 7).

**Table 6:**
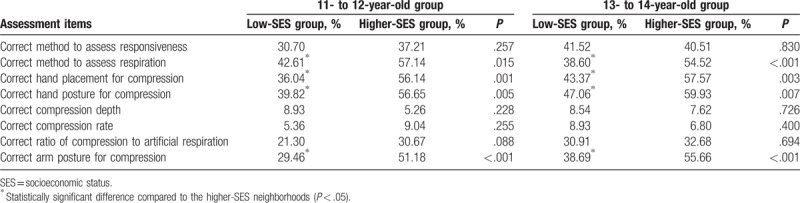
Results of the pretraining questionnaires stratified according to neighborhood socioeconomic status and age.

**Table 7:**
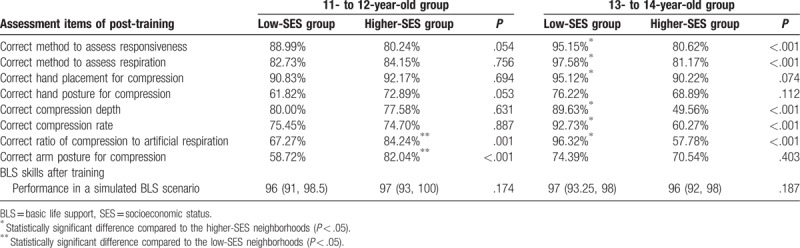
Results of the post-training questionnaires and BLS skills after training stratified according to neighborhood socioeconomic status and age.

## 4 Discussion

This study revealed that primary and secondary school children in China had little pretraining knowledge of CPR. However, with training, there was a significant improvement in the basic theory and skills of CPR. Students from low-SES neighborhoods had less pretraining knowledge of CPR. However, their performance was similar with students from higher-SES neighborhoods on the post-training questionnaire and the skills assessment, and better among students aged 13 to 14 years.

Bystander CPR training for children is essential, as it ensures that they will have the skills to act in an emergency. This would strengthen safety in the community and improve cardiac survival rates. Furthermore, bystander CPR training may satisfy future employment requirements. Primary and secondary school children are focused, curious for knowledge, and motivated to learn new skills. In addition, they are likely to share newly acquired information with their parents and friends. Therefore, training student bystander CPR would result in more people acquiring BLS skills in the long term.

In the present study, we found a significant improvement in all aspects of bystander CPR after the theoretical training program. These findings were especially pertinent to the knowledge of the depth, the rate for compression, and the ratio of compression to artificial respiration. And the percentage of them was increased from 7.58% to 72.94%, 8.18% to 72.57%, and 28.89% to 76.55%, respectively. Our findings suggest that Chinese primary and secondary school children could effectively learn bystander CPR theoretical knowledge by training. Previous studies have confirmed that bystander CPR learning could increase the awareness of first aid and improve outcomes of OHCA.^[1,6,10,11,23]^

In fact, the students were not confident performing practical bystander CPR on the manikin after the theoretical training in our study. Therefore, practical training was conducted subsequently. After guided-practice, 92.84% of school children performed bystander CPR proficiently and 82% of school children had an excellent performance (scoring >90 points on a scale of 0–100 points). In accordance with these findings, previous studies have shown that school children in Europe and North America are able to acquire BLS skills after practical training.^[10,11,24]^ These results show that theoretical combined with practical training is a feasible and effective method for primary and secondary school children in China.

In our study, scores on topics such as the compression depth, rate, and the ratio of chest compression to artificial respiration were low. These findings are in accordance with the results of the previous study.^[13]^ This situation may suggest that Chinese primary and secondary school students have little pretraining knowledge of CPR and an urgent need for first aid knowledge.

In addition, our study showed that students from low-SES neighborhoods had less CPR knowledge before training and worse performance on pretraining questions. The continuous urbanization and industrialization has caused the labor force from low-SES neighborhoods to enter cities, leaving their children behind with few resources at home in China. Such differences may be due to the lack of public health education and the shortage of medical resources in low-SES neighborhoods. Students from low-SES neighborhoods were not willing to share the CPR knowledge with others (27.91% vs. 20.85%, *P* < .05), which may be attributed to insufficient knowledge and no confidence. After training, 13 to 14 years old students from low-SES neighborhoods had the best performance on the post-training questionnaire. All the students from the low-SES neighborhoods, independent of age, did as well as ones from higher-SES neighborhoods on total scores for the skills assessment. As rural children comprise approximately 30% of China's pediatric population, the present study highlights the importance of bystander CPR education and training in schools in low-SES neighborhoods in China

Lack of knowledge of BLS skills, fear of performing bystander CPR incorrectly, and concerning about legal liability associated with poor outcomes following the administration of bystander CPR may be the main reasons for low initiating bystander CPR.^[15]^ This suggests that legislative support is essential if bystander CPR training programs are to be universally popularized in China.

## 5 Limitations

This study was associated with several limitations. First, the generalizability of the findings to other regions in China remains unknown. Second, although the study demonstrated immediate improvements in knowledge and skills of bystander CPR, mastery requires retraining and practice regularly. Third, the ethnic factors, immigrants and the students who did not present on the day of training were not considered. Further studies aimed at developing bystander CPR training programs suitable for younger children are warranted.

## 6 Conclusions

School children in China have a poor pretraining knowledge of bystander CPR. However, with training, there was a significant improvement in the basic theory and skills. Bystander CPR training efforts should be targeted to Chinese primary and secondary school children, especially in low-SES neighborhoods.

## Acknowledgments

The authors thank the Zhejiang University Medical College Clinical Skills Training Center for providing training teachers and training models.

## Author contributions

**Conceptualization:** Xiangming Fang, Xu Shen, Yan Wang.

**Data curation:** Xu Shen, Haihong Wang, Xia Xu.

**Formal analysis:** Yaqi Sun, Xia Xu.

**Investigation:** Hui Li, Lihua Chu. Jialian Zhao, Ya Wang, Haihong Wang, Hui Ye.

**Methodology:** Baoli Cheng, Yan Wang.

**Project administration:** Xiangming Fang, Hui Li.

**Writing – original draft:** Hui Li, Hui Ye.

**Writing – review & editing:** Yan Wang, Guohao Xie, Xiangming Fang.

## Supplementary Material

Supplemental Digital Content

## References

[1] Böttiger BW Van Aken H Training children in cardiopulmonary resuscitation worldwide. Lancet 2015;385:2353. 10.1016/S0140-6736(15)61099-6 26088639

[2] Nichol G Thomas E Callaway CW Regional variation in out-of-hospital cardiac arrest incidence and outcome. JAMA 2008;300:1423–31. 18812533 10.1001/jama.300.12.1423 PMC3187919

[3] Pan J Zhu JY Kee HS A review of compression, ventilation, defibrillation, drug treatment, and targeted temperature management in cardiopulmonary resuscitation. Chin Med J (Engl) 2015;128:550–4. 25673462 10.4103/0366-6999.151115 PMC4836263

[4] Tanigawa K Iwami T Nishiyama C Are trained individuals more likely to perform bystander CPR. An observational study. Resuscitation 2011;82:523–8. 21354688 10.1016/j.resuscitation.2011.01.027

[5] Swor R Khan I Domeier R CPR training and CPR performance: do CPR-trained bystanders perform CPR? Acad Emerg Med 2006;13:596–601. 16614455 10.1197/j.aem.2005.12.021

[6] Böttiger BW Aken HV Kids save lives: training school children in cardiopulmonary resuscitation worldwide is now endorsed by the World Health Organization (WHO). Resuscitation 2015;94:A5–7. 26209417 10.1016/j.resuscitation.2015.07.005

[7] Chamberlain DA Hazinski MF European Resuscitation Council, et al.. Education in resuscitation: an ILCOR symposium: Utstein Abbey: Stavanger, Norway: June 22–24, 2001. Circulation 2003;108:2575–94. 14623795 10.1161/01.CIR.0000099898.11954.3B

[8] Lockey AS Georgiou M Children can save lives. Resuscitation 2013;84:399–400. 23328405 10.1016/j.resuscitation.2013.01.011

[9] Neumar RW Eigel B Callaway CW American Heart Association response to the 2015 Institute of Medicine Report on strategies to improve cardiac arrest survival. Circulation 2015;132:1049–70. 26130121 10.1161/CIR.0000000000000233

[10] Beck S Meier-Klages V Michaelis M Teaching school children basic life support improves teaching and basic life support skills of medical students: a randomised, controlled trial. Resuscitation 2016;108:1–7. 27576085 10.1016/j.resuscitation.2016.08.020

[11] Abelairas-Gómez C Rodríguez-Núñez A Casillas-Cabana M Schoolchildren as life savers: at what age do they become strong enough? Resuscitation 2014;85:814–9. 24614187 10.1016/j.resuscitation.2014.03.001

[12] Hill K Mohan C Stevenson M Objective assessment of cardiopulmonary resuscitation skills of 10–11-year-old schoolchildren using two different external chest compression to ventilation ratios. Resuscitation 2009;80:96–9. 18952356 10.1016/j.resuscitation.2008.08.005

[13] Huang Q Hu C Mao J Are Chinese school children willing to learn and perform bystander cardiopulmonary resuscitation? J Emerg Med 2016;51:712–20. 27727043 10.1016/j.jemermed.2016.02.033

[14] Shi HT Ge JB Improving public defibrillator use in China. Lancet 2016;388:1156–7. 27650087 10.1016/S0140-6736(16)31609-9

[15] Chen M Wang Y Li X Public knowledge and attitudes towards bystander cardiopulmonary resuscitation in China. Biomed Res Int 2017;2017:3250485. 28367441 10.1155/2017/3250485 PMC5359437

[16] Steenland K Henley J Calle E Individual- and area-level socioeconomic status variables as predictors of mortality in a cohort of 179,383 persons. Am J Epidemiol 2004;159:1047–56. 15155289 10.1093/aje/kwh129

[17] Roux AVD Merkin SS Arnett D Neighborhood of residence and incidence of coronary heart disease. N Engl J Med 2001;345:99–106. 11450679 10.1056/NEJM200107123450205

[18] Fosbøl EL Dupre ME Strauss B Association of neighborhood characteristics with incidence of out-of-hospital cardiac arrest and rates of bystander-initiated CPR: implications for community-based education intervention. Resuscitation 2014;85:1512–7. 25180920 10.1016/j.resuscitation.2014.08.013

[19] Dahan B Jabre P Karam N Impact of neighbourhood socio-economic status on bystander cardiopulmonary resuscitation in Paris. Resuscitation 2017;110:107–13. 27865747 10.1016/j.resuscitation.2016.10.028

[20] Perkins GD Handley AJ Koster RW European Resuscitation Council guidelines for resuscitation 2015: section 2. Adult basic life support and automated external defibrillation. Resuscitation 2015;95:81–99. 26477420 10.1016/j.resuscitation.2015.07.015

[21] Wissenberg M Lippert FK Folke F Association of national initiatives to improve cardiac arrest management with rates of bystander intervention and patient survival after out-of-hospital cardiac arrest. JAMA 2013;310:1377–84. 24084923 10.1001/jama.2013.278483

[22] Chiang WC Ko PC Chang AM Bystander-initiated CPR in an Asian metropolitan: does the socioeconomic status matter? Resuscitation 2014;85:53–8. 24056397 10.1016/j.resuscitation.2013.07.033 PMC4023511

[23] Takei Y Kamikura T Nishi T Recruitments of trained citizen volunteering for conventional cardiopulmonary resuscitation are necessary to improve the outcome after out-of-hospital cardiac arrests in remote time-distance area: a nationwide population-based study. Resuscitation 2016;105:100–8. 27263486 10.1016/j.resuscitation.2016.05.021

[24] Plant N Taylor K How best to teach CPR to schoolchildren: a systematic review. Resuscitation 2013;84:415–21. 23246989 10.1016/j.resuscitation.2012.12.008

