# Supplementary material for: Bystander cardiopulmonary resuscitation training in primary and secondary school children in China and the impact of neighborhood socioeconomic status: A prospective controlled trial
Source: Medicine (Baltimore). 2018 Oct 5;97(40):e12673. doi: 10.1097/MD.0000000000012673 (PMC6200495; doi:10.1097/MD.0000000000012673)
Supplement: Supplemental Digital Content [file medi-97-e12673-s001.docx]

Supplementary Appendix A: The scoring system for our CPR skills assessment which was developed from the 2015 European Resuscitation Council Guidelines and the Cardiff Test for BLS and Automated External Defibrillation Version 3.1: Assessment Guidelines [20–21].

**Supplementary Appendix A**

| **Steps** | **Procedures** | **10 marks for each step is perfectly done** |
| --- | --- | --- |
| **1** | Check for consciousness and call for help |  |
| **2** | Place the patient in a proper position |  |
| **3** | Hand placement for chest compression |  |
| **4** | Rate of chest compressions (100-120 times/minute) |  |
| **5** | Depth of chest compression (5-6cm) |  |
| **6** | Compression technique (Two elbows locked, compressing straight down with the heel of one hand and the heel of the other hand placed atop and with fingers of both hands lifting above the chest) |  |
| **7** | Method to clear the air way / open the air way |  |
| **8** | Way of artificial ventilation |  |
| **9** | Ratio of compression to ventilation |  |
| **10** | Proficiency |  |
|  | Remarks |  |

The scoring system for CPR skills

Supplementary Appendix B: The CPR questionnaire for pre-training and post-training which was designed according to a previous research [22], while considering the actual situation of China, and were distributed in Chinese.

**Supplementary Appendix B**

**CPR questionnaire**

**一、general information**

Gender: Age: Grade:

1. **Awareness of CPR and willingness of sharing (Please only choose one answer for each question)**
2. Have you heard of cardiopulmonary resuscitation?

Never

Yes

If yes, by what?

1. TV; (2) Newspapers; (3) internet; (4) Others
2. Have you ever received CPR training?
3. No
4. Yes

If yes, how long did it take?

(1) One year; (2) two years; (3) three years and over three years

1. If you have mastered CPR knowledge, are you willing to share it with others?
2. No
3. Yes (who would you like to? ____________)

**三、The theory knowledge of Cardiopulmonary resuscitation**

1. What is most common reason for cardiopulmonary arrest in daily life?

(1) Electric shock; (2) Poisoning; (3) Sudden Cardiac Arrest; (4) Trauma

2. How do you judge if the person is unconscious?

(1) Shout loud, shake his/her shoulder gently

(2) Shout loud, shake his/her shoulder

(3) Shout, shake his/her shoulder gently

(4) Shout, shake his/her shoulder

1. How to judge if a person has spontaneous respiration?
2. Check for chest motion; (2) Listen to breath sound; (3) Feel the breath on your cheek and ear;
3. where is the correct position for chest compression?

(1) In the sternum between the two nipples; (2) On the Cardiac apex; (3) Upper part of the sternum; (4) On the left border of the sternum and in the 5th intercostal space

1. The method of cardiac compression?

(1) Compression with the heel of one hand with fingers lifting above the chest; (2) Compression with five fingers of one hand; (3) Compression with the heel of one hand and the heel of the other hand placed atop and fingers of both hands lifting above the chest; (4) Compression with fingers from two hands overlapping

1. Depth of cardiac compression?
2. Half of the anteroposterior diameter of the chest; (2) 2-3 cm; (3) 4-5 cm; (4) 5-6cm
3. How often is the frequencies of cardiac compression?

(1) 60-80 times/minute; (2) 80-100 times/minute; (3) 100-120 times/minute ;(4) above120 times/minute

1. For rescuing an adult, what is the ratio of compression to ventilation?
2. 15:1; (2) 15:2; (3) 30:1; (4) 30:2
3. What is correct way of compression?

(1) Two elbows bent, compressing hard with the arms; (2) Two elbows bent, compressing with the help of your upper body weight and muscles of the shoulders and arms; (3) Two elbows locked, compressing hard straight down with arms; (4) Two elbows locked, compressing straight down with the help of your upper body weight and of muscles of the shoulders and arms

1. when ventilating, do you need to hold the person’s nose?
2. Yes
3. No
